# Historical experience in the elimination of visceral leishmaniasis in the plain region of Eastern and Central China

**DOI:** 10.1186/2049-9957-3-10

**Published:** 2014-03-20

**Authors:** Li-Ren Guan, Zhong-Xing Wu

**Affiliations:** 1 National Institute of Parasitic Diseases, Chinese Center for Disease Control and Prevention, Shanghai 200025, PR China; 2 Jiangsu Institute of Parasitic Diseases, Wuxi 214064, China

**Keywords:** Visceral leishmaniasis, Elimination, Plain region, Eastern and Central China

## Abstract

Visceral leishmaniasis (VL) (*kala-azar*) was most seriously prevalent in the plain regions of eight provinces/municipalities in the eastern and central parts of China. In the early 1950s, the number of counties/cities endemic for VL and the number of cases in the plain regions accounted for 60% and 80%, respectively, of the total numbers in the entire country. By implementing comprehensive control measures, including treatment of patients for eliminating the source of infection and spraying insecticide in endemic villages to kill sandflies, VL transmission has been brought under control in this region by the early 1960s, and no new infected cases have been found since 1983, achieving the goal of eliminating VL.

## Multilingual abstracts

Please see Additional file 1 for translations of the abstract into the six official working languages of the United Nations.

## Review

Visceral leishmaniasis (VL) was one of the most serious parasitic diseases endangering people’s health in China. It was highly prevalent in vast rural areas north of the Yangtze River, especially in the plain regions of the Shandong, Jiangsu, Anhui, Henan, and Hebei provinces. Since the local peasants lived in poor conditions at that time, most of the patients could not afford treatment, resulting in the continuous transmission and spread of the disease. Since the founding of the People’s Republic of China, it was confirmed through extensive investigations, that VL was prevalent in 16 provinces/autonomous regions/municipalities with an estimated number of 530,000 patients in 1951.

By establishing professional institutions/stations for disease control in endemic provinces/prefectures, conducting experimental studies on the methods for prevention and treatment, mobilizing medical staff in rural areas, and launching control activities in the early 1950s, the number of patients decreased yearly, and the VL epidemic has been brought under control in the plain region of eight provinces/municipalities in the early 1960s. No new human infection has been found since 1983, reaching the goal of eliminating VL.

At present, VL is still prevalent, or in sporadic distribution, in six provinces/autonomous regions, the average annual number of cases is not more than 450, and most of the cases are distributed in the west hilly and desert areas. Considering that the epidemiological factors of local VL are still not fully understood and the vector sandfly is exophilic, it is still necessary to develop effective preventive methods.

## History of VL in China

It is difficult to verify when VL first appeared in China and when the VL epidemic occurred. Wang CJ had consulted a lot of historical literature written since 1271 by famous doctors of Chinese traditional medicine in VL endemic areas. He found records of cases with similar signs/symptoms of VL in the “Rushishanfang Medical Records” (1885–1895), written by Gao Yingqing, a doctor of Chinese medicine in Huai’an city in the period of Emperor Guangxu’s rule (1875–1908) during the Qing Dynasty [1]. Because the main symptom was “Pi-Kuai” or “Pi” (splenomegaly) in the left abdomen, and it was an epidemic situation, Gao named this disease “Yi-Pi” (an infectious disease with hepatosplenomegaly). In the records, Gao described the disease’s clinical manifestation as follows: “In recent years, Yi-Pi has become prevalent. There were no records of this disease in previous medical books. It was induced by the resistance of the liver and the spleen to a pathogenic factor. “Pi” is usually found under the left rib, and patients in a serious condition have decayed gums or abdominal swelling, with most patients eventually dying.” According to the clinical manifestations, where the illness arose, and the epidemic situation described by Gao, the so-called “Yi-Pi” was undoubtedly VL. In 1935, the Qingjiangpu VL research team was engaged in VL control activities, and was informed by the local senior residents that cases with similar signs/symptoms as VL were frequently seen in the rural areas 50 years earlier (i.e. about 1885) [2]. This information confirmed that “Yi-Pi” was indeed VL. Therefore, it can be inferred that VL was prevalent in north Jiangsu at least as early as the 1880s.

The first VL patient who acquired the infection in China (and was parasitologically confirmed) was a German soldier who joined the eight-power allied forces invasion to China in 1900. The soldier entered Beijing through Qingdao, and later returned home due to illness, dying in Leipzig in 1902. By section examination of the liver, spleen, and marrow during the autopsy, many corpuscles were found in the macrophages. It was not until 1903, when *Leishmania* was discovered, that it was understood that the corpuscles found in the tissue section of the soldier was *Leishmania*; therefore, his death might have been due to VL. Considering that he got infected in the region of Shandong and Hebei, the presence of VL was confirmed for the first time in China [3]. Thereafter, parasitologically diagnosed VL cases were found successively in Hubei [4], Shandong [5], Tianjin and Beiping (Beijing) [6,7], and Henan [8]. The VL epidemic in China was now verified.

## The epidemic situation

Before 1935, most of the information about the VL epidemic status and its geographical distribution in China was acquired from the case reports of medical institutions. Up until 1935, an investigation team, formed by the bureau of civil affairs of the Jiangsu province, conducted VL surveys in north Jiangsu. The results indicated that VL has been prevalent in 14 counties in the region since 1927. In a village with only a hundred people in the Siyang County, 30 VL cases were found. In the city of Suqian, 20 VL cases were found among the 60 students of the Lailongji primary school [9]. According to the investigation made by the Qingjiangpu VL research team in the Huaiyin District, VL infection was found in 82% of the local villages. By conducting house-to-house surveys in 15 villages, the infection rate by household was found to be as high as 83.3%; the number of VL cases accounted for 10.7–31.9% of the total population in those villages [2]. In the Shandong province, VL was also severely prevalent. Based on an investigation, 223 VL cases were found in nine counties in east Shandong in 1945, and the number of cases increased to 4,110 in 1947 [10]. In 1933, 23 VL cases were admitted to hospital in the Anyang County, Henan province; the case number increased to 111 in 1934 [11]. Between October 1946 and May 1947, the number of patients hospitalized in Kaifeng reached 1,111 [12].

After the victory of the Anti-Japanese War in 1945, domestic scholars reviewed literature on VL in the country in the period between 1904 and 1946. Their review indicated that VL was widely distributed in 211 counties/cities in 13 provinces north of the Yangtze River, namely Jiangsu, Anhui, Hebei, Shandong, Henan, Hubei, Liaoning, Rehe (now in the north Hebei province and the Ningcheng Country of Inner Mongolia), Sichuan, Shaanxi, Gansu, Xikang (now in the areas of Ya’an of the Sichuan province), and Xinjiang [13].

**Figure 1:**
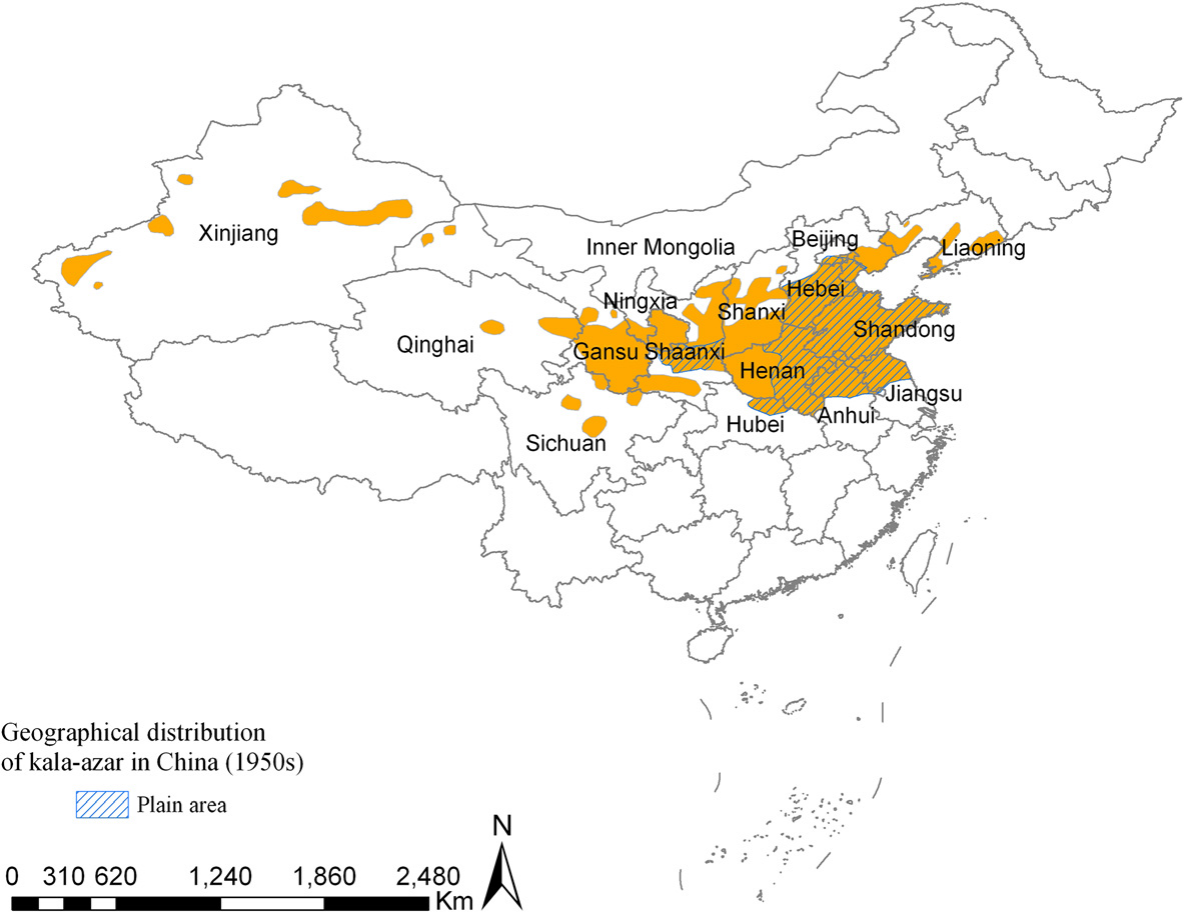
Geographical distribution of visceral leishmaniasis in China (1950s).

With the founding of the People’s Republic of China in 1949, an epidemiological survey on VL was carried out between 1951 and 1953 due to cooperation between professional institutions concerned with VL control and the relevant anti-epidemic stations. The results confirmed that there was a VL epidemic or sporadic distribution in 650 counties/cities of 14 provinces/municipalities north of the Yangtze River, namely Liaoning, Beijing, Hebei, Shandong, Henan, Jiangsu, Anhui, Hubei, Shaanxi, Gansu, Ningxia, Sichuan, Qinghai, and Xinjiang. Thereafter, VL prevalence was also confirmed in Shanxi (1957) and western Inner Mongolia (1972) (see Figure 1). Based on the survey, in 1951, it was estimated that the VL prevalence rate in the 14 provinces/municipalities was about 10–50 per ten thousand, and there were at least 530,000 VL patients in the entire country that year [14].

Even though VL is widely distributed, the heaviest endemic areas of VL were in the plain region of the central and eastern parts of the country, the North China Plain, which covers Beijing, Hebei, Shandong, Henan, Jiangsu, Anhui (32°–40°N, 114°–121°E), and the Central Shaanxi Plain. It was also frequently seen in some counties/cities in the northern fringe of the Jianghan Plain of the Hubei province. On these plains, about 395 counties/cities had VL transmission, accounting for 60.8% of the total endemic counties/cities in the whole country (395/650). Except for Beijing and Hebei–where no statistical data were available–the number of cases in the other six provinces could have been over 400,000 (see Table 1), causing great damage to people’s health, and agricultural and industrial production. Therefore, VL control activities were firstly carried out in the central and eastern plain regions.

**Table 1:** Epidemic situation of VL in the plain region

***Province***	***Year of survey***	***Number of endemic counties/cities***	***Prevalence rate (/10,000)***	***Estimated number of cases (10,000)***
Shandong [15]	1951	135	35.0	18.0
Jiangsu [16]	1951–1952	24	44.0	3.2
Anhui [16]	1951–1952	19	18.0	2.5
Shaanxi [17]	1952	44	46.0	4.0
Henan [18]	1951	79	50.0	12.2*
Hebei [19]	1950–1953	88	-	**
Hubei [20]	1957	6	5.8	0.0508

*In that year, there were 122 counties/cities with presence of VL and 180,000 estimated patients. Calculated by the average number of cases, there were at least 122,000 VL cases in 79 counties/cities in the plain region of the province.

**Before the founding of the People’s Republic, there were about 100,000 VL cases [19].

## Control activities

### Administrative measures

#### Establishment of professional institutions for VL control in major endemic areas

Between 1950 and 1953, anti-VL stations were established in Shandong, north Jiangsu, north Anhui, Henan, Hebei, and Shaanxi to carry out VL control work. The main tasks were to:

– Determine the epidemic situation and epidemiological factors of VL;

– Train technical personnel for VL control at different levels, and establish an anti-VL network from county to township level; and

– Supervise, inspect, and give technical guidance to VL control.

After 1955, the institutions of VL control in the provinces were merged with other institutions of parasitic disease control, but they still kept a separate department for VL control.

#### Formulation of the VL control plan

An implementation plan for VL control was developed at the National Conference on Epidemic Prevention led by the Ministry of Health in 1951. In order to strengthen technical guidance, the East China Branch of the National Institute of Health (predecessor of the National Institute of Parasitic Diseases, China CDC) was authorized to work in cooperation with the institutions working in VL control in endemic provinces to carry out VL control and research, starting in the Shandong province. In 1957, based on the “National Program of Agricultural Development” (draft) issued in 1956 (which set out to eliminate the most severe diseases endangering people within a period of 12 years starting from 1956), the Ministry of Health issued a document on “the basic situation of VL and the requirement of VL control”, and set a goal to effectively control VL throughout the country before 1962. With the issuance of this document, the administrative departments of health in the relevant provinces/autonomous regions/municipalities strengthened their efforts in VL control. Meanwhile, the Ministry of Health requested that experts compile a “Manual for VL control” and distribute it to the institutions/stations as guidelines.

#### Production and supply of therapeutic drugs

In 1949, pentavalent sodium antimony gluconate was synthesized and six kinds of preparations (20–100 mg pentavalent antimony/1 ml of water solution) were provided by domestic drug research institutes for trials to treat VL. To facilitate the standards and quality control, the Ministry of Health entrusted the Shandong Xinhua Pharmaceutical Factory to be responsible for the production and supply of pentavalent antimony (100 mg/1 ml) in 1950, which fully met the needs for VL treatment in the entire country.

#### Provision of free VL treatment services

The government decided to provide anti-VL drugs free of charge to all VL patients. The patients were responsible for the cost of treatment of complications in principle, but if they could not afford it, the government would provide them subsidies. Since 1955, free insecticides were provided for sandfly control by the VL control institutions, and village doctors responsible for VL treatment would get a service subsidy from governments based on the number of cases treated.

### Studies on control strategies

#### Investigation on the epidemic situation of canine visceral leishmaniasis (CVL) and its relationship with VL

In the 1930s–1940s, 2,888 domestic dogs were surveyed with microscopic examination of iliac puncture smear to find *Leishmania* in the rural areas of three counties with VL transmission in the Jiangsu, Hebei, and Shaanxi provinces. Only four dogs were found to have the CVL infection [2,21,22]. Between 1951 and 1956, along with extensively conducting VL control activities in the country, a survey of infected dogs was conducted in 53 endemic counties/cities of VL in seven provinces, in order to find out the prevalence of CVL and its role in human VL transmission. A total of 50,486 domestic dogs were examined. The results showed that in those provinces, except for the Central Shaanxi Plain where relatively larger numbers of CVL were found, the VL rather than the CVL epidemic was more severe. The results also indicated that in the majority of the villages with VL patients, no infected dogs were found, and the canine infection rate in VL endemic areas was much lower than in humans (see Table 2), showing that a local VL epidemic was not closely related to CVL (the VL patient was the main source of infection). Therefore, prompt detection and complete cure of VL cases to eliminate the source of infection should be the key strategy against VL transmission in the plain region.

**Table 2:** Canine visceral leishmaniasis in the plain region of the seven provinces studied

***Province***	***Year of survey***	***No. of counties surveyed***	***No. of dogs examined***	***No. of sick dogs (/10000)***	***No. of counties with CVL***	***Human VL prevalence in the same period (/10000)***
Shandong [23]	1951–1952	21	22,342	15 (0.7)	8	31
Jiangsu [2]	1936	1	35	0	0	319
Jiangsu [23]	1951–1952	6	8,439	0	0	44
Anhui [23]	1951–1952	7	6266	1 (0.2)	1	18
Hebei [22]	1939–1942	1	1,780	4 (2.7)	1	34
Hebei [24]	1956	5	1125	0	0	-
Henan*	1953–1955	3	1040	1	1	-
Xian, Shaanxi [21]	1944–1945	1	1073	4 (3.7)	1	40
Central Shaanxi Plain [17]	1952–1953	8	11,095	11 (10.0)	5	35
Hubei [20,25]	1957	3	179	0	0	5.8

*Henan Provincial Institute of Visceral Leishmaniasis Control, unpublished data, 1956.

A total of 3,562 stomach blood specimens of *Phlebotomus chinensis* (the vector of VL) were collected from 13 counties in the plain region of Shandong, Jiangsu, Anhui, and Shaanxi provinces, to determine the blood source of sandfly by precipitin test. The results indicated that all the *Ph. chinensis* samples had the blood-sucking habit on man and big livestock, and of the 1,843 blood specimens collected from Tai’an (in Shandong), 29 (1.6%) showed a positive reaction to dogs’ antiserum [26]. Therefore, it is understandable why very few CVL cases or no CVL cases were found–the VL prevalence might be related to the blood-sucking habit of the sandfly.

#### Development of therapeutic regimes with homemade pentavalent sodium antimony gluconate

Trivalent antimony tartar emetic was tried in the treatment of VL in China in the 1910s, and was found to be highly toxic and less effective [27-29]. In the 1930s, it was replaced by pentavalent antimony, and the drugs trial-used were Neostibosan [30,31], Urea Stibamine [32], Solustibosan [33], and Pentostam [34,35]. Although their cure rate reached 95–97%, it was still difficult to use pentavalent antimony extensively because the first two drugs had a relatively high toxic level for humans, had to be prepared immediately before injection due its unstability in water solution, and was usually not suitable for intramuscular injection. The latter two drugs were less toxic and could be used for intramuscular injection. However, because all four chemicals had to be imported from abroad, they were not affordable for most people.

In 1950, the Shandong Xinhua Pharmaceutical Factory synthesized pentavalent sodium antimony gluconate (trade name: Stibii hexonas). It was a concentrated aqueous solution which could be used for intravenous or intramuscular injection. It was suitable for rural medical staff to use and therefore played an important role in VL control.

Before large-scale use of homemade pentavalent sodium antimony gluconate, clinical trials were firstly conducted in Shandong and other places to determine its therapeutic effect and the method suitable for its application in rural areas.

(1) *Six-day therapy:* The total dosage was calculated according to body weight (see Table 3), equally divided into six daily doses, and given by either intravenous injection or intramuscular injection. Between 1950 and 1953, over 4,000 VL cases were treated like this with a speedy effect. Fifteen days after the completion of the treatment course, the body temperature in most patients returned to normal, appetite increased, spleen reduced, white cell count increased to over 6,000, red blood cells and hemoglobin also increased markedly, and the parasite clearance rate reached 99.0%. Only 1% of the total patients failed the treatment or died of complications; among 3,897 cases observed for over two years after treatment, 3,570 cases (91.6%) were cured, while 289 (7.4%) relapsed. If calculating by combining with the number of cured cases in the second treatment course, the cure rate reaches 97.4% [36,37]. The six-day therapy was used as the therapeutic scheme of choice in the endemic areas.

**Table 3:** Total dosages of homemade pentavalent sodium antimony gluconate

***Body weight (kg)***	***Volume of drug solution (ml)***	***Amount of pentavalent antimony (mg)***
5–10.0	12–18	1200–1800
10.1–15.0	18–24	1800–2400
15.1–20.0	24–30	2400–3000
20.1–25.0	30–36	3000–3600
25.1–30.0	36–42	3600–4200
30.1–50.0	42–48	4200–4800
>50	48–54	4800–5400

(2) Three-day therapy: The total dosage is again calculated according to body weight (see Table 3), divided equally into six injections, twice daily, in the morning and afternoon, at an interval of six to eight hours, completed in three days. Between 1950 and 1951, 265 cases were treated in Shandong with no severe side effects or accidental deaths. Ten days after completion of the treatment, the parasite clearance rate reached 99.6%. Among the 114 cases followed up for 1½–2 years, four patients relapsed, one patient died, and the cure rate was 96.5% [38]. Expanded application showed that the three-day therapy was suitable for patients in good health and without complications.

(3) Three-week therapy: The total dosage for children was 200 mg/kg body weight, each dose was 33.3 mg/kg body weight; the total dosage for adults was ⅔ of the children’s dosage, two injections per week, completed in three weeks. In Beijing, 117 cases were followed up for over 11 months after treatment; no relapse occurred [39]. Between 1954 and 1957, 90 cases were treated at the Shandong Provincial Institute for VL Control, and the parasite clearance rate was 97.3% one month after completion of the treatment. Over two years’ follow-up observation showed that seven patients (7.8%) relapsed, one case died of heart disease four months after treatment, and the cure rate was 91.0%. This therapy was suitable for those in poor health or with serious illness.

#### Phlebotomus chinensis identified as the VL-transmitting vector in the plain region

Between 1926 and 1951, investigations and experimental studies were conducted in Shandong, Jiangsu, Beiping, and other places. It was confirmed that *Ph. chinensis* was the vector transmitting VL in Central and Eastern China. This finding was based on: (1) *Ph. chinensis* was the main or the sole sandfly species in these endemic areas of VL. (2) After the laboratory-bred *Ph. chinensis* feeding on VL patients and CVL dogs or hamsters infected with VL, the promastigote of *Leishmania* could multiply rapidly in large numbers in the digestive tracts of sandflies and move to the pharynx and the proboscis [40-43]. (3) In the VL endemic villages, *Ph. chinensis* naturally infected with promastigotes could be found [44]. When the promastigotes were injected into the abdominal cavity of a healthy hamster, the hamster would get the VL infection [45]. (4) When *Ph. chinensis*, which was experimentally or naturally infected with the *Leishmania* from CVL fed on a healthy hamster after its gastric blood was completely digested, the hamster could get VL [46,47]. Thereafter, the promastigotes isolated from the stomach of *Ph. chinensis* caught in the endemic area in Sichuan were examined with immunoassay, and were identified as *Leishmania donovani*[48]. The above results were completely identical to the World Health Organization’s (WHO’s) six indicators for identifying the transmission vector of *Leishmania*[49].

#### Experimental study on sandfly control

The bionomics of sandflies were investigated in Shandong, north Jiangsu, and north Anhui between 1951 and 1953. The results demonstrated the following characteristics of *Ph. chinensis* in the plain region in respect to its bionomics [50]:

– Its activity season is as short as 3½ months and most sandflies only had one generation a year.

– The lifecycle is as long as 50 days from egg to adult, and most larvae would enter the diapause period in late August and wait until the following May to emerge into adult sandflies.

– *Ph. chinensis* is endophilic, staying in the room at daytime and keeping active at night on the inside and outside wall surfaces of residential areas. This indicates that sandflies can keep contact with the insecticide-sprayed wall for a long time.

– The investigation in Shandong showed that the breeding places of the larvae were widely distributed and scattered, hence it was hard to control effectively.

Based on the above-mentioned bionomic characteristics of *Ph. chinensis*, a strategy taking adult sandfly control as the main measure for interrupting the role of *Ph. chinensis* in VL transmission was suggested.

In collaboration with the East China Institute foe VL Control, the East China Branch of National Institute of Health conducted a field trial on *Ph. chinensis* control with chemical insecticides in the highly endemic township of Tai’an, Shandong, in 1951. A wettable powder (available from the markets) or emulsion of DDT and gammexane were prepared into suspension and sprayed on the walls of farmhouses once several days before the activity season of adult *Ph. chinensis*, and at the beginning or before the peak of the activity season. The dosage used for pure DDT was 1.05–2.24 g/m^2^ and for gamma isomers gammexane, it was 0.11–0.17 g/m^2^. When the two insecticides were used in combination, the dosage was half of that when used alone. Two methods were adopted: one was the whole village spraying on the inside and outside wall surfaces and roofs of their houses; the other was spraying on all inside wall surfaces and the roof. In 1952–1953, however, the spraying was performed on all inside wall surfaces and on part of the roofs in the entire village. After spraying, besides continuing the observation of sandfly density, the residual effect of the insecticide was also observed by examining the rate death of sandflies after exposure to the sprayed walls and determining the residual amount of insecticide on the wall surfaces.

The observation on the sandfly density in different experiment areas demonstrated that the whole village spraying with various formulations of DDT and gammexane was remarkably effective against sandfly. After spraying, not only did sandflies disappear in the first year, but also few sandflies could be found in the villages for several years after. The residual effect of DDT on the walls lasted two years. A six-year longitudinal observation after spraying showed that sandfly was still rare in the village. The residual effect of gammexane was only one to two months. In the following year, sandfly would appear in the village, but the density increased very slowly to the level similar to the control areas during the sixth year after spraying.

Since 1953, the practice of the whole village spraying insecticide against *Ph. chinensis* was also carried out in north Jiangsu, north Anhui, and other areas. The results of the observations in the first year of spraying and the following two to three years showed that sandflies were rarely found, similar to the result in Shandong.

Considering that the activity range of *Ph. chinensis* was quite limited in the plain region, an experimental study on sandfly control with insecticide spraying in patients’ homes was carried out. Ten households were selected in the city of Tai’an in Shandong, the Huaiyin District of Jiangsu, and the Huaiyuan County of Anhui in 1954. In the first year of spraying, only a few sandflies were occasionally found in the households sprayed, and the sandfly density in different households was lower than in the control areas the following year [51,52].

#### Observation on the effect of case treatment in combination with sandfly control in VL control

In the Budong township of Tai’an, Shandong, 12 villages were selected for VL control measures combining patient treatment with the practice of the entire village spraying insecticide successively between 1951 and 1953. Since 1955, no new cases were found in the 12 villages [52]. Thereafter, either clinical treatment of the cases or an integrated control measure was conducted in another 90 villages in the city of Tai’an. The results showed that even though case treatment alone could reduce the incidence yearly, a combined measure of chemotherapy with sandfly control brought VL incidence down and reached the goal of control or elimination of the disease more rapidly (see Table 4) [15].

**Table 4:** Effect of case treatment alone or as an integrated control measure combining case treatment with sandfly control

***Year***	***Control measure I***	No. of VL cases in 45 villages*	***Control measure II***	***No. of VL cases in 45 villages *** [15]
1952	—	—	Treatment of clinical cases	207
1953	—	—	Treatment of clinical cases	198
1954	Treatment of clinical cases	95	Treatment of clinical cases	103
1955	Treatment of clinical cases	84	Treatment of clinical cases and spraying in patients’ homes	104
1956	Treatment of clinical cases	77	Treatment of clinical cases and spraying in patients’ home	45
1957	Treatment of clinical cases	60	Treatment of clinical cases and spraying in patients’ home	29
1958	Treatment of clinical cases and whole village spraying	56	Treatment of clinical cases and spraying in patients’ home	8
1959	Treatment of clinical cases	5	—	—
1960	Treatment of clinical cases	1	—	—
1961	—	0	—	—

*Data by Wang CJ and Shao QF from the symposium on the current situation of VL by the National Institute of Parasitic Diseases in 1974.

In view of the good effect of insecticide spraying in Shandong, Anhui, and Jiangsu against *Ph. chinensis*, specific recommendations for expanded implementation were put forward in 1955. There were as follows [52]: (1) The minimum dosage for spraying pure DDT was 1.5 g/m^2^ and 0.12 g γ/m^2^ of gammexane. (2) In areas with relatively high transmissions, whole village indoor spraying should be conducted. In areas with less patients or sporadic distribution, spraying patients’ homes could be adopted, but the area of spraying should be expanded to the households within a range of 13–15 m around each of the VL patient’s home. (3) The appropriate time for spraying was mid- or late May, i.e. the beginning of the sandfly activity season. The Ministry of Health issued a document to the provincial health departments of Shandong, Jiangsu, Anhui, Henan, Hebei, Shaanxi, and Gansu asking them to supervise the implementation of this by their professional institutions for VL control.

## Outcomes of the control strategies

Between 1950 and 1958, spleen palpation, inquiry of disease history, serological or parasitological examination on suspected VL cases, free treatment of confirmed cases in the township clinics, and treatment of severe patients in the county hospital or competent institutions at the higher level were performed by professional VL control institutions as the core, with participation from rural health workers in the endemic areas. The number of cases treated was 248,427, 53,820, and 110,999 in Shandong, Shaanxi, and Jiangsu, respectively. Since 1955, with mass examination and treatment, sandfly control with insecticide spraying was simultaneously conducted. Due to the extensive use of pesticides for farming at that time, the range of insecticide spraying was expanded. By combining treatment of patients to eliminate the source of infection with insecticide spraying to kill the sandflies, VL prevalence in the plain region of the provinces declined remarkably by 1958; from 35/10000 in 1950 to 0.3/10000 in Shandong [15], from 50/10000 in 1951 to 0.77/10000 in Henan [18], from 44/10000 in 1950 to 1.0/10000 in Jiangsu [53], and from 46/10000 in 1952 to 1.1/10000 in Shaanxi (central plain region) (Xue JD, personal correspondence 1973), indicating that VL transmission was brought under control.

Considering that few VL cases could be found in the endemic area, the Shandong province adopted different measures between 1960 and 1968. This included treating the confirmed cases by grassroots health workers conducting household interviews and necessary examinations, checking the sandfly density in the villages where patients were found, and spraying insecticide in the patients’ homes. Due to this, the number of cases further decreased. The VL prevalence in this province decreased from 0.3/10000 in 1958 to 0.05/10000 in 1963, and then to 0.001/10000 in 1970 [54]. In Henan, the VL prevalence decreased to 0.1/10000 in 1966 [55]. After 1983, no new indigenous cases were found in Shandong, Jiangsu, Anhui, Henan, Hebei, Hubei, and Central Shaanxi Plain, revealing that VL was virtually eliminated in these areas. The time and place of the onset of the infection and confirmation of the last VL case in the provinces are listed in Table 5. In addition, only small endemic areas with a few cases were recorded in the provinces of Ningxia, Qinghai, and Liaoning. In the 1970s, it was declared that VL was eliminated after integrated control activities.

**Table 5:** The last VL case in the plain regions of the seven provinces studied

***Province***	***County***	***Year of disease onset of the last case***	***Year of the case confirmed***	***Reference***
Shandong	Shanxian	1973	1986	Teng *et al.*, [56]
	Gaomi	1973	1991	Zhang *et al.*, [57]
Jiangsu	Xinyi	1959	1976	East regional cooperation meeting, 1976
Anhui	Xiuxian	1969	1969	Xiuxian County Anti-epidemic Station (Kong XC, 2002, personal correspondence)
	Funan	1969	1972	East regional cooperation conference, 1976
Henan	Xuchang	1983	1983	Wang *et al.*, [58]
Hebei	Dingxian	-	1978	Guan *et al.*, [59]
Hubei	Junxian	1959	1963	Gui *et al.*, [60]
Shaanxi (central plain region)	Meixian	1965	1965	Meixian County Anti-epidemic Station, 1973

- means onset time unknown.

## Surveillance

After 1958, VL prevalence in the human population decreased significantly, and surveillance began instantly in some provinces. The surveillance covered three aspects, namely follow-up survey of the clinical cases to grasp the epidemic dynamics, leishmanin intradermal test to immunologically evaluate the trend of the local transmission, and survey of the sandfly density to evaluate whether transmission was interrupted.

Since 1958, the Shandong province began to pay attention to the surveillance of VL patients. At first surveillance took place to manage the distribution of therapeutic drugs and the medical units at different levels were asked to report to the local anti-epidemic station or institute of parasitic disease control once they found a suspected VL case, made parasitological examination of the case for confirmation, and treated the case with sodium antimony gluconate. After 1970, blood samples were collected on filter paper from the suspected patients in all localities and mailed to the provincial institute of parasitic disease control for IFAT, or to the staff of the institute, to make further examinations in order to confirm the diagnosis and then give treatment. Between 1972 and 1987, 18 VL cases were detected in 16 counties/cities before 1971 [61]. In 1991, one case was found in another county, and the year of onset was 1973 [57]. Thereafter, no new cases have been found.

In 1989, a leishmanin intradermal test was conducted successively on 10,239 rural inhabitants from 78 townships of 37 counties/cities previously with different prevalence of VL. The positive rate of the skin test increased in accordance with the age of the testees, being 0 (0/8020) for the group aged 1–29, 1.25% (14/1124) for the group aged 30–39, 6.5% (37/568) for the group aged 40–49, and 9.3% (47/507) for the group ≥50 years old [54]. This phenomenon exactly reflected the changing process of VL prevalence until 1959 when VL was brought under control.

Between 1973 and 1988, professional workers went to the previous endemic areas to conduct a sandfly survey during the peak season of sandfly density; a total of 334 natural villages in 144 townships of 62 counties/cities were investigated. *Ph. chinensis* was found in 51 natural villages, the number was only 0.5 sandfly per man-hour, and the largest number of sandflies was 23 found in a village by a hill. In 18 villages where VL cases were detected before 1972, no sandflies were found [61]. The above results demonstrated that VL was eliminated in the Shandong province.

The VL prevalence in the Henan province decreased to 0.77/10000 in 1958. During the period between 1973 and 1982, a total of 645 suspected cases were found in the entire province. Through parasitological examination, only five were confirmed as VL, distributed between four counties, with medical history showing that none of them were new infections and one of them was an imported case from Shanxi [55]. In 1983, another case was detected in Xuchang, in which the patient got onset of *kala-azar* that year [58]. Between 1984 and 1990, IFAT or parasitological examination was conducted on 350 suspected cases found through preliminary screening in different counties; all were excluded from VL [18]. In the previous endemic areas of VL (three counties), 767 dogs were examined and none were found to be infected [18,55].

In 1976, a leishmanin intradermal test was performed on 466 inhabitants in a village where one case was confirmed as VL; only two people who were infected with VL 30 years ago and have been cured for a long time showed a positive reaction [55].

The surveillance on *Ph. chinensis* density began in 1973 and was completed in 1996. No sandflies were found in the east Henan plain region, but in 18 out of the 20 counties surveyed in the west Henan hilly region, some were still found [62].

The VL prevalence in the Hubei province was relatively light with a limited endemic area than that of the plain regions of other provinces, and there was no new epidemic report after 1963. In order to check the effect of the control activities, the Hubei Provincial Institute of Parasitic Disease Control conducted physical examination on 11,520 people in five previous VL endemic counties/cities between 1975 and 1976, and no VL cases were found. In 1978, 517 inhabitants of the above two counties were examined again with a leishmanin intradermal test; only 26 people (5.0%) born before 1958 showed a positive reaction [60]. Between 1997 and 1999, the previous endemic areas were further investigated; only one imported case from Xinjiang was detected. Skin tests were performed on 13,890 people in the previous endemic areas; only one among the 162 people born before transmission interruption showed a positive reaction. By indoor and outdoor inspection in 60 villages, 1,208 sandflies were collected and all were identified as *Sergentomyia*, which was unrelated to VL transmission. The result of the surveillance indicated that VL was eliminated from this province [63].

In the northern Jiangsu province, VL prevalence declined to about 1/10000 in 1958 [64], and only 53 cases were detected in 1960. During the mass survey in 1973, 11 cases were found and their onset time of VL was before 1965 [65]. Between 1991 and 1992, 56 townships/towns in 14 previous VL endemic counties/cities were further investigated; earlobe blood samples of 1,017 people were collected for IFAT. All showed a negative reaction and no sandflies were found [53].

In 1996, a leishmanin intradermal test was performed on 2,572 farmers from three counties in northern Anhui province, where VL was heavily prevalent in the 1950s. The test results indicated that 774 under 19 years of age all had a negative reaction, and the positive rate in the age group 20 years and above was 9.5% (174/1828). In addition, all those who had a positive reaction were above 23 years old [66], indicating that VL transmission had been interrupted over 20 years ago.

Although active surveillance was not conducted in the Central Shaanxi Plain and the central and south plain of Hebei, no new infections were found after 1965 and 1978, respectively, revealing that they had reached the goal of eliminating VL.

## Conclusion

Epidemiologically, VL in China can be divided into three types, namely anthroponotic in the plain region, anthropozoonotic in the hilly region, and enzootic in the desert region [67,68]. The route of transmission for anthroponotic type is VL patient → sandfly → healthy people. The incidence in human population was highest in the endemic areas, could cause an epidemic, the majority of infected people were children over five years of age and young people with a feature of family clustering, CVL was rare, and *Ph. chinensis* was endophilic. In view of these characteristics, adopting the strategy of timely detection and treatment of patients for eliminating the source of infection and spraying insecticide indoors to kill sandflies, VL in this type of endemic region had been brought under control by the early 1960s. Thereafter, surveillance on human VL and the transmitting vector was conducted, and necessary intervention measures were taken. Since 1983, no new human infection has been found for 30 years, reaching the WHO criteria of disease elimination. Sufficient supply of homemade highly-effective, low toxic therapeutic drugs for VL; development of a suitable therapeutic regime; and implementation of effective sandfly control ensured the elimination of VL. The establishment of professional institutions by the government and relevant people-benefiting policies gave important support to disease elimination.

With VL elimination in the central and eastern plain regions, the endemic areas of VL reduced greatly in China, from over 650 counties/cities in 16 provinces/autonomous regions/municipalities in 1950 down to 30 counties/cities in six provinces/autonomous regions in the northwest including Xinjiang, Gansu, Shaanxi, Sichuan, Shanxi, and Inner Mongolia in the 1980s; the number of VL patients in the whole country decreased from 530,000 in 1951 to 360 in 1990 [69], showing a remarkable achievement.

The above-mentioned six provinces/autonomous regions with VL transmission or a sporadic distribution were mostly hilly and desert areas. The existing problems are: in parts of the hilly region, dogs are raised by most farmers which causes an increase of CVL and human VL; due to population movement, VL cases might be imported in some counties/cities originally without VL transmission, thus likely to form new endemic areas [70]. In people without symptoms or a history of a *Leishmania* infection, PCR or ELISA has been used to detect *Leishmania*-specific DNA or antibody, and it has been found that a number of asymptomatic *Leishmania* infections existed in the local human population and dogs. The role of asymptomatic infection in local VL transmission remains to be studied [71,72]. In the desert area, because the wild animal host remains to be elucidated and the vector sandfly is exophilic, a suitable control strategy is still not available at present. In recent years, local outbreak of VL has occurred in villages in south Xinjiang, and the epidemiological factors are unknown [73]. The problem of VL control in the above two types of regions remains to be investigated and solved.

## Competing interests

The authors wish to declare that they have no competing interests.

## Authors’ contributions

LRG and ZXW collected the data and wrote this paper. Both authors read and approved the final manuscript.

## Supplementary Material

Additional file 1 Multilingual abstracts in the six official working languages of the United Nations. Click here for file
