# Supplementary material for: Historical experience in the elimination of visceral leishmaniasis in the plain region of Eastern and Central China
Source: Infect Dis Poverty. 2014 Mar 20;3:10. doi: 10.1186/2049-9957-3-10 (PMC4022322; doi:10.1186/2049-9957-3-10)
Supplement: Additional file 1 — Multilingual abstracts in the six official working languages of the United Nations. [file 2049-9957-3-10-S1.pdf]

Translation of the abstract into the six official working languages of the United Nations

خبرة تاريخية في التخلص من داء الليشمانيات الحشوي (Visceral leishmaniasis (VL في المنطقة السهلية من شرق ووسط الصين.

لي- رن جُوان ، وزهونج-كسينج وُو.

الملخص: مرض الليشمانيات الحشوي (VL) ، أو كالا آزار (الحمى السوداء) كان سائداً بشكل أكثر خطورة في المنطقة السهلية لثمانية أقاليم/بلديات في الجزئين الشرقي والأوسط من الصين. وفي أوائل الخمسينات (من القرن العشرين - 1900) ، بلغ عدد المقاطعات/المدن اللتي تُعد موطناً لداء الليشمانيات الحشوي (VL) ، وعدد حالات الإصابة به في المناطق السهلية بلغ 60% و 80% على التوالي من العدد الإجمالي في القطر كاملاً. وبتنفيذ إجراءات للسيطرة الشاملة ، بما في ذلك معالجة المرضى للتخلّص من مصدر العدوى ، ورش المبيدات الحشرية لقتل ذُبَاب الرَّمل (الفواصِد) في القرى الموطنية ، أمكن السيطرة على انتقال/انتشار داء الليشمانيات الحشوي (VL) في هذه المنطقة بحلول أوائل الستّينات ، ولم يُعثَر على حالات جديدة للإصابة به منذ العام 1983 ، متحققاً بذلك هدف التخلّص من داء الليشمانيات الحشوي (VL).

Translated from English version into Arabic by Lotfi Abdolhaleem, through

TRANSLATORS
WITHOUT BORDERS

中国中、东部平原地带消除内脏利什曼病的历史经验

管立人，吴中兴

摘要

中国中、东部 8 省/直辖市的平原地带原来是内脏利什曼病流行最为严重的地区，1950 年代初期，该地带有内脏利什曼病流行的县/市数及病例数分别约占全国综述的 60%和 80%。经过治疗患者以消灭传染源和病村喷洒杀虫剂灭蛉的综合措施，1960 年代初即控制了该类地带内脏利什曼病的流行，1983 年以后即未再出现新感染病例，达到了消除的目标。

Translated from English version into Chinese by Guan Li-ren, through

Retour d'expérience historique sur l'élimination de la leishmaniose viscérale dans les régions de plaine de l'est et du centre de la Chine

Li-Ren Guan, Zhong-Xing Wu

Résumé : La prévalence de la leishmaniose viscérale (ou *kala-azar*) était très préoccupante par le passé dans les régions de plaine de huit provinces ou municipalités dans l'est et le centre de la Chine. Au début des années 1950, les circonscriptions et villes où elle était endémique dans les régions de plaine représentaient, respectivement, 60 % et 80 % du total pour le pays entier. Grâce à des mesures complètes de lutte contre la maladie (traitement des patients en vue d'éliminer la cause de l'infestation, pulvérisation d'insecticides dans les villages d'endémie pour éliminer les phlébotomes), la transmission de la leishmaniose viscérale a été jugulée dans ces régions au début des années 1960. Le but d'élimination de la leishmaniose viscérale est atteint, puisque aucun cas nouveau d'infection n'a été recensé depuis 1983.

Translated from English version into French by Suzanne Assenat, through

TRANSLATORS
WITHOUT BORDERS

Исторический опыт ликвидации висцерального лейшманиоза в низменных районах Западного и Центрального Китая

Ли-Рен Гуан, Чжун-Зинг Ву

Краткое изложение: Висцеральный лейшманиоз (ВЛ) (*кала-азар*) был наиболее широко распространен в низменностях восьми провинций/муниципалитетов в западных и центральных областях Китая. В начале 1950-х гг. количество областей/городов, в которых ВЛ был эндемичным, и количество случаев заболеваний в низменных районах составляли 60% и 80% соответственно от общего количества случаев по всей стране. Благодаря внедрению необходимых контрольных мер, включая лечение пациентов для уничтожения источника инфекции и распыление инсектицидных средств в эндемичных деревнях для уничтожения москитов, распространение ВЛ в этом регионе находилось под контролем с начала 1960-х гг., и новых случаев инфицирования не наблюдалось с 1983 г.. Таким образом, была достигнута цель полной ликвидации ВЛ.

Translated from English version into Russian by Elena McDonnell, through

TRANSLATORS WITHOUT BORDERS

Experiencia histórica en la eliminación de la leishmaniosis visceral en las llanuras del este y el centro de China

Li-Ren Guan, Zhong-Xing Wu

Resumen: La leishmaniosis visceral (LV), o *kala-azar*, estaba gravemente extendida en las llanuras de ocho provincias/municipalidades de las zonas orientales y centrales de China. A principios de los años 50, el número de provincias/ciudades en las que la LV era endémica y el número de casos en las regiones llanas alcanzaba respectivamente el 60 % y el 80 % del número total en todo el país. Con la aplicación de medidas de control exhaustivas, como tratar a los pacientes para eliminar la fuente de infección y fumigar los poblados endémicos con insecticida contra la mosca de arena, la transmisión de LV quedó controlada en la región a principios de los años 60, sin que hayan aparecido casos nuevos de infección desde 1983. Se ha alcanzado así el objetivo de eliminar la LV.

Translated from English version into Spanish by Maira Belmonte, through

TRANSLATORS
WITHOUT BORDERS
